# Medical Society of the District of Columbia

**Published:** 1871-01

**Authors:** 


### MEDICAL SOCIETY OF THE DISTRICT OF COLUMBIA.

This Society held its 53d annual meeting on the 2d of the present month. W. P. Johnson, M. D., upon retiring as President for the year 1870, made a handsome valedictory. The following officers were elected for the ensuing year:

President, Dr. J. M. Toner; Vice Presidents, Drs. S. C. Busey and Wm. Marbury; corresponding secretary, Dr. W. B. Drinkard; recording secretary, W. W. Johnson; treasurer, Dr. F. A. Ashford; librarian, A. F. A. King; board of examiners, Drs. W. G. Palmer, D. R. Hagner, Lewis Mackall, jr., B. Thompson, C. M. Ford; censors, C. H. Leiberman, J. F. Thompson and Thomas Miller.

Dr. Toner, upon taking the Chair, made an interesting address.

